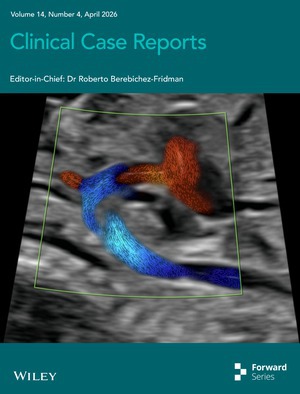# Cover Image

**DOI:** 10.1002/ccr3.72479

**Published:** 2026-04-09

**Authors:** Sarah van den Wildenberg, Andrea Stoop‐Berends, Ingrid M. van Beynum, Bas R. Rebel, Attie T. J. I. Go, Eric A. P. Steegers, Jérôme M. J. Cornette

## Abstract

The cover image is based on the article *Blood Speckle Imaging as a Novel Adjunct in the Prenatal Assessment of Congenital Heart Disease: Insights From Aortic Arch Pathology and Septal Defects* by Sarah van den Wildenberg et al., https://doi.org/10.1002/ccr3.72163.